# Retrospective exploratory evaluation of individual pigs’ behaviour involved in tail biting during rearing and fattening

**DOI:** 10.1371/journal.pone.0316044

**Published:** 2025-01-13

**Authors:** Karen Kauselmann, E. Tobias Krause, Hansjörg Schrade, Lars Schrader

**Affiliations:** 1 Friedrich-Loeffler-Institut, Institute of Animal Welfare and Animal Husbandry, Celle, Germany; 2 Bildungs- und Wissenszentrum Boxberg (LSZ), Boxberg, Germany; CREA: Consiglio per la ricerca in agricoltura e l’analisi dell’economia agraria, ITALY

## Abstract

Tail biting is one of the biggest welfare problems in pigs. However, depending on the individuals involved (e.g., tail biter/victim), pigs seem to change their behaviour prior to tail biting events, which raises the possibility of early detection and thus prediction and prevention of tail biting. In this retrospective explorative study, we used datasets from four different studies with 9 trials of rearing (4 pens/trial with 24 pigs/pen) and fattening (8 pens/trial with 12 pigs/pen) that focused on the exploration behaviour of undocked pigs towards plant-based enrichment materials. From this dataset, we identified 8 pens from rearing (n = 192 pigs) and 6 pens from fattening (n = 72 pigs) in which individual tail biters were identified. From this dataset, we investigated whether any *a priori* behavioural changes in exploration or feeding could be identified with respect to tail biting. Furthermore, the effects of weight parameters from suckling to fattening were examined. Using linear mixed effects models, we found that exploration duration was linked to days prior to tail biting in rearing, depending on CatPig (category of pigs: biter, victim, neutral pig) (P = 0.001), in fattening independent of CatPig (P<0.0001), and by duration, amount and frequency of feed consumption in fattening (P<0.0001). Some weight parameters covaried with CatPig in rearing (weight-gain suckling: P = 0.0018; weaning weight: P = 0.019) and fattening (weaning weight: P = 0.07; start weight at fattening: P = 0.03; weight-gain rearing: P = 0.02). Suitable indicators for future early detection trials of tail biting could be exploration duration in rearing and fattening and feeding data in fattening. Moreover, weight parameters in rearing and fattening and exploration duration in rearing may be used to identify individual pigs that might become tail biters in an upcoming tail biting event. The retrospective explorative nature of our analysis revealed interesting patterns; however, further studies are needed to confirm our findings.

## Introduction

Tail biting is considered one of the most important welfare problems in conventional pig housing systems, i.e., with fully slatted floors and bare pens. When exploration behaviour cannot be adequately satisfied, it seems that it is often redirected against pen mates, which can result in tail biting [1]. However, tail biting is a behavioural disorder with multifactorial causes and thus can also be caused by other triggers [2, 3]. Both pig-related factors, e.g., behaviour, genetics and breed, growth, health and sex, and housing environment-related factors, e.g., climate, enrichment material, feeding, management, space availability and group size, can affect the prevalence of tail biting [4]. Offering appropriate enrichment materials can reduce tail biting by encouraging pigs to perform species-specific exploration behaviours, i.e., rooting, chewing and nosing [5, 6]. In particular, plant-based enrichment materials fulfil the recommended characteristics, as they are changeable, manipulable, chewable, edible or odorous, increasing exploration behaviour in pigs [1, 7–12]. However, owing to practical problems with the slurry system of pens with slatted floors, these plant-based enrichment materials are often not used. Thus, to avoid tail biting, tails are docked. Although routine tail docking is prohibited by law [13] and tail docking cannot completely avoid tail biting [14–16], approximately 77% of pigs in the European Union have docked tails [17]. The consequences of tail biting range from minor wounds associated with pain for the pig to secondary diseases, reduced weight gain or even death of affected animals [16, 18, 19].

Early detection of tail biting outbreaks would be a helpful tool for farmers to reduce or even avoid both economic losses and reduced welfare by preventing tail biting and better managing pigs with undocked tails. Prior to a tail biting outbreak, pigs are reportedly more active [20–23], and biters show a higher enrichment manipulation than control pigs do [23]. Furthermore, during tail biting outbreaks, victims show reduced feed consumption [24, 25]. In addition, daily weight gains during rearing affect the occurrence of tail biting [26], and tail biters have been found to have reduced weight gains during suckling [27]. Thus, behavioural changes such as exploration behaviour and feed consumption or even weight gains in an earlier stage of a pig’s life might indicate tail biting outbreaks or even potential tail biters prior to injurious tail biting. In most studies, time-intensive video analyses or live observations have been used to evaluate behaviours that could predict tail biting, which is not very suitable for farmers to use in practice. In addition, the quality of the video image often does not allow for the discernment between the behaviour of individuals and therefore the detection of biters [20]. Systems that automatically and individually record the behaviour of pigs could offer a solution for less time-consuming evaluations of potential tail biting, indicating behaviours that might also be feasible for farmers [28]. One method that provides promising results in automatically recording the activity levels of individual pigs is the use of ultrahigh-frequency radiofrequency identification (UHF-RFID) systems [e.g. 11, 29–32]. One difference between the RFID systems currently available is that UHF-RFID has the fastest data transmission rate compared with low-frequency (LF) and high-frequency (HF) RFID systems. This allows several animals (i.e., UHF-RFID tags on the pig’s ear) to be detected simultaneously with one UHF-RFID antenna. Thus, this system enables evaluations of automatically recorded data on the behaviour (i.e., time pigs stay near the antenna) of individual animals in the group simultaneously. However, as tail biting is a multifactorial problem [2, 3], the use of further data, e.g., automatically recorded feed consumption, for which LF-RFID can be used (i.e., to identify and record data of single animals at single feeder stations), is recommended. Another possibility for finding possible indicators for tail biting predictions and identifying biters is the use of already routinely recorded weights of individual pigs (e.g., birth weight or weaning weight), for which no additional workload would be needed.

In this retrospective explorative analysis, we used data from four published studies [11, 12, 29, 33] to investigate whether changes in automatically UHF-RFID-recorded exploration duration of pigs and LF-RFID-recorded feed consumption can be observed and potentially used in the future to predict tail biting outbreaks in rearing and fattening pigs. Usually, tail biting is recognized on commercial farms at the pen level, i.e., when an outbreak has started and spread to a certain level throughout the group. To a certain extent, this was also the case in the four previously published studies, but on the basis of coincidental reports, a substantial number of individual tail biters could be identified. These cases were analysed here in detail. We hypothesized that there are differences in exploration durations and feed consumption among tail biters, victims and neutral pigs prior to tail biting outbreaks and that low weaning weights and reduced daily weight gains during an earlier stage of life covary with an increased prevalence of becoming a tail biter.

## Methods

The data used for this retrospective explorative study originate from previously published studies that addressed different research questions (i.e., independent of the research question of this retrospective explorative study) and for which an ethical statement can be found in the respective published paper (see Table 1). The animals used in the studies on which this retrospective explorative study is based were kept on a licenced farm (VVVO-Number: 08 128 0140 538) in accordance with German legislation [34] at the time of data collection, and the pigs were marketed after the studies. The animals had more space and more enrichment material than the minimum requirements according to German law, and no interventions were carried out on the animals. For this reason, no approval was required for this retrospective explorative study.

**Table 1:** The offered enrichment materials, change intervals of the enrichment material and references for detailed information on the studies from which the datasets were obtained for the retrospective analyses.

Study	Enrichment material	Interval of enrichment material change	Reference (for detailed information)
1	Straw pellets	Biweekly	[11]
	Lucerne pellets		
	Chopped straw		
	Chopped hay		
2	Flavoured straw pellets	Weekly	[12]
	Fried onion		
	Strawberry		
	Ginger		
	Almond		
	Vanilla		
	Water (control)		
3	Chopped straw	Continuously (no change)	[29]
	with maize kernels		
	without maize kernels		
4	Chopped straw	Continuously (no change)	[33]
	once daily		
	four times daily		

**Fig 1:**
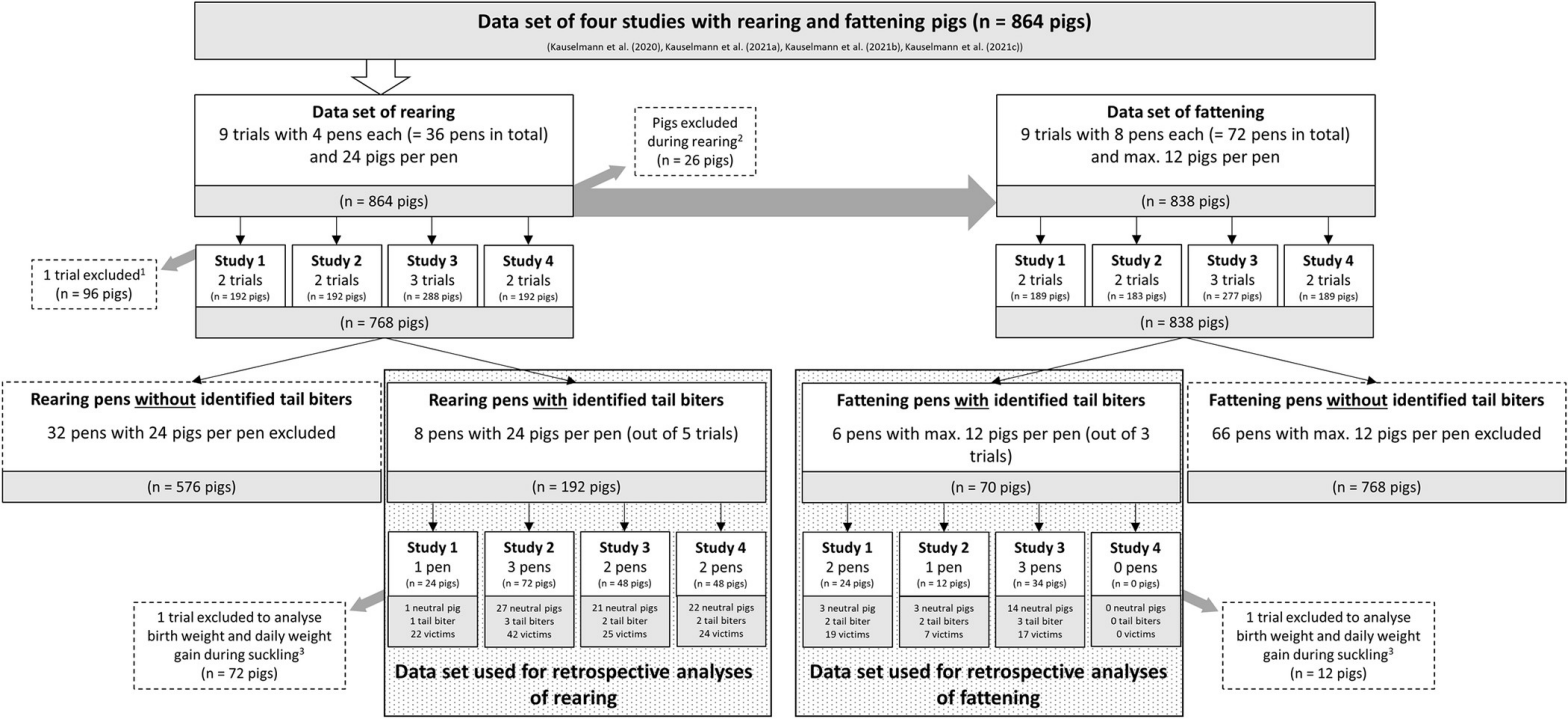
Overview of the available dataset from four previous studies with the respective number of rearing and fattening pigs (grey boxes), excluded data (dashed boxes) and data used for the retrospective analyses (boxes with a dotted background). The grey arrows show the movement of pigs from rearing to fattening during the study period and the removal of pigs. ^1^ Data excluded due to technical issues. ^2^ Pigs were excluded during rearing because of health issues (n = 18 pigs) or because they were identified as tail biters (n = 8 pigs). ^3^ Pigs were excluded because of missing birth weights (see S1 Table for further information).

For this retrospective explorative study, we considered the exploration data of four previously published studies that focused on RFID-based measurements of exploration behaviour towards different plant-based enrichment materials [11, 12, 29, 33]. Thus, a total of 864 pigs with undocked tails (see Fig 1) from nine trials during rearing (four pens with 24 piglets per trial) and nine trials during fattening (eight pens with 12 pigs per trial) were used. These studies took place at the same location, i.e., “Bildungs- und Wissenszentrum Boxberg (LSZ)”, Germany, and the settings between studies were almost identical except for the type of plant-based enrichment material offered and the intervals of enrichment material change or refilling of the material dispenser (Table 1). In the four previously published papers, tail biting was analysed for those instances for which actions against tail biting had to be applied (i.e., tail biting animals or bleeding tails were observed). Therefore, for our retrospective analysis, we distinguished among three retrospectively assigned categories of pigs (CatPig), i.e., biters, victims and neutral pigs (i.e., pigs identified as neither a biter nor a victim), to investigate behavioural differences between the pigs in these categories (categorization described below). For this retrospective explorative study, only pens in which individual pigs were identified as tail biters were used for analyses in rearing and fattening. Thus, for retrospective evaluations of rearing and fattening, different datasets were used, which were analysed separately (see Fig 1). Tail biting also occurred in the pens not included in the retrospective evaluation. However, as biters in these pens could not be individually identified, they were not included so that the result would not be distorted by the incorrect categorization of the individual pigs. In general, the number of biters in the present study is likely to be underestimated. Furthermore, we also considered individual weights and weight gains from all pigs of the considered pens over the entire production period (i.e., suckling, rearing and fattening).

### Rearing: Animals and housing

**Fig 2:**
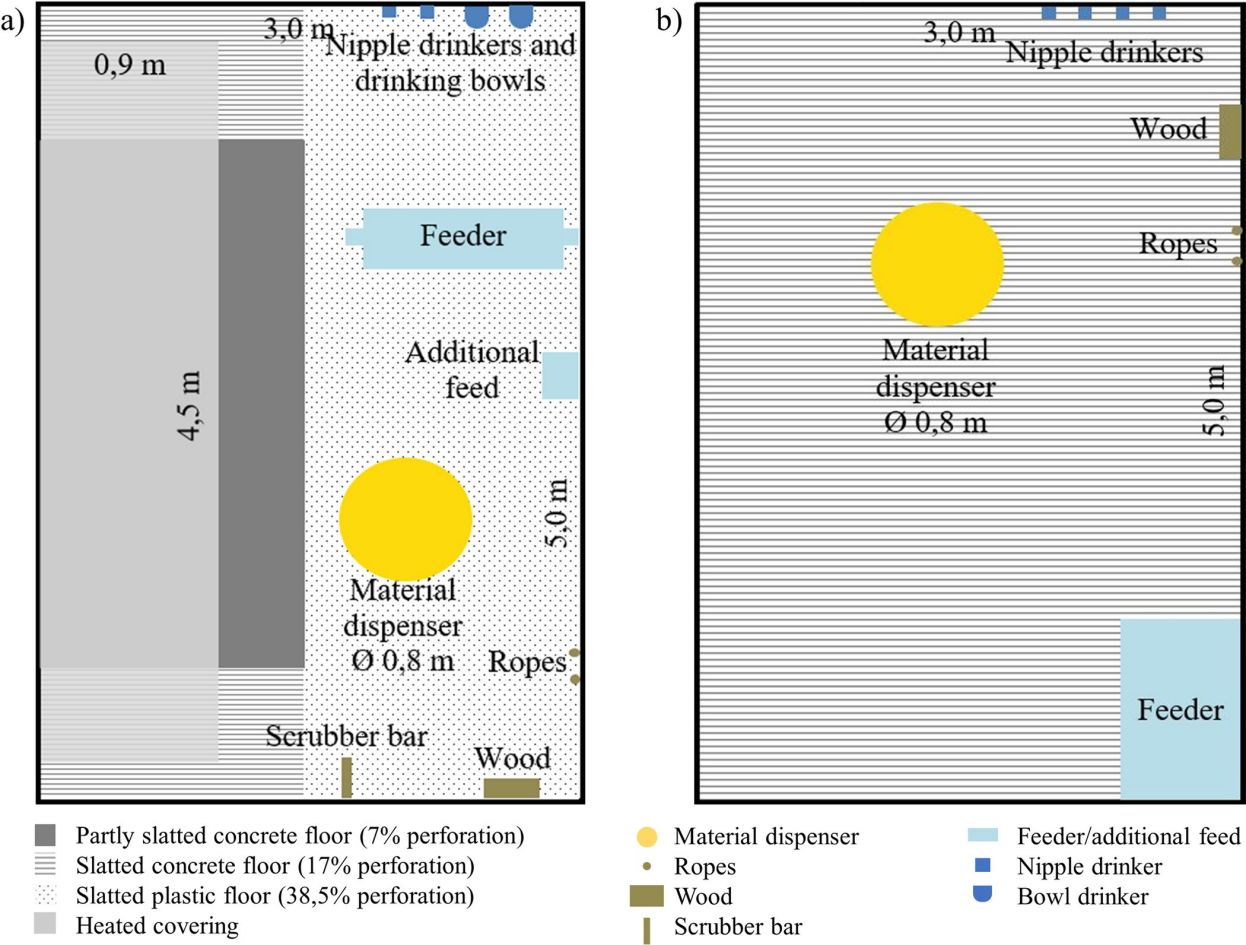
Schematic drawing of a) a rearing pen and b) a fattening pen with a partly slatted concrete floor with 7% perforation (dark grey), a slatted concrete floor with 17% perforation (lined), a slatted plastic floor with 38.5% perforation (dotted) and a heated covering (transparent grey).

**Fig 3:**
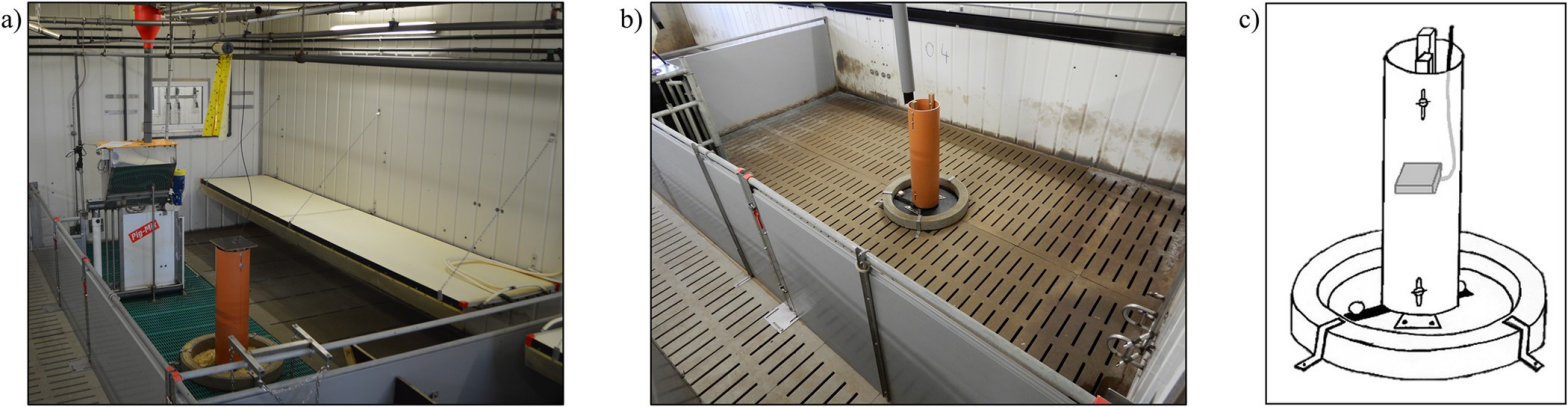
a) Rearing pen with a material dispenser, b) fattening pen with a material dispenser and c) drawing of the material dispenser with a UHF-RFID antenna (drawing reused with permission from Elsevier (licence number: 5638130812474)).

We identified eight individual tail biters, one in each of eight rearing pens, in five out of the nine abovementioned trials during rearing (see Fig 1). Thus, all further analyses in rearing were performed with this subset of eight pens in which a tail biter was identified in a total of 192 crossbred piglets (German Piétrain × German Hybrid) with mixed sexes (100 females and 92 castrated males) (Fig 1). Individual identified tail biters were removed from pens, and further action was applied to the remaining animals in the pen (see below). The average weight at birth from all the considered pigs was 1.6 kg (± 0.3 kg SD). Since the birth weights of one trial were not collected, only data from 120 pigs could be considered for analyses of birth weight and weight gain during suckling (see S1 Table). At weaning at an age of four weeks, all considered piglets (i.e., only piglets with intact tails) weighed an average of 7.7 kg (± 1.6 kg SD) and were equipped with one unique numbered UHF-RFID tag (MS Tag Round UHF, MS Schippers, Bladel, Netherlands) per ear for individual identification at the material dispenser (described below). After weaning, the 96 piglets per trial were housed in a forced ventilated barn, where they were divided into four identical rearing pens (24 piglets per pen) with 15 m^2^ (5 m × 3 m) floor space (Figs 2A and 3A). The floors consisted of a 7.5 m^2^ slatted plastic floor (38.5% perforation), a 4.5 m^2^ partly slatted concrete floor (7.0% perforation) under a heated covering and a 3.0 m^2^ slatted concrete floor (17.0% perforation). All pens in all four studies were identically equipped with two sisal ropes, a scrubber bar, a piece of wood hanging on a metal chain and a material dispenser (see description below). All pens were continuously numbered throughout all trials with a unique PenID. Piglets had *ad libitum* access to water from two nipple drinkers and two drinking bowls per pen and to mashed feed with an automatic feeder (animal:feeding place ratio 2.4:1). The feed composition changed after two weeks of rearing (see S2 Table). These feeding procedures were identical in all the trials. During the last week of rearing, piglets additionally received pellet concentrate (25 kg per pen) to prepare them for the change of feed in fattening. After seven weeks of rearing at an age of eleven weeks, the pigs considered for analyses in rearing weighed 30.6 kg (± 5.4 kg SD) on average. At this time, data collection in the rearing pens stopped, and the pigs were moved to the fattening pens (described below). All the piglets were treated identically among all the trials except for the contents of the material dispensers (see Table 1).

### Fattening: Animals and housing

A total of seven tail biters could be identified (see description below) in six fattening pens in three of the nine abovementioned trials (see Fig 1). In Study 2, one tail biter was miscategorized (i.e., categorized as “removed due to illness” instead of “removed due to tail biting”), which was corrected for this retrospective evaluation. We used a total of 70 crossbred fattening pigs (German Piétrain × German Hybrid) with mixed sexes (34 females and 36 castrated males) for analyses in fattening. The pigs used for analyses during fattening had a birth weight of 1.50 kg (since the birth weights of one trial were not collected, only data from 58 pigs could be considered for analyses regarding birth weight and weight gains during suckling) and a weaning weight of 7.51 kg (Fig 1 and S1 Table). For fattening, the pigs from one rearing pen were randomly divided into two fattening pens (12 pigs per pen). At the beginning of fattening, the considered pigs weighed 30.6 kg (± 5.4 kg SD) on average. Individual identified tail biters were removed from pens in all studies, and further action was applied to the remaining animals in the pen (see below). The fattening pens had fully slatted concrete floors (17% perforation) and 15 m^2^ of floor space (Figs 2B and 3B). All pens were identically equipped with two sisal ropes, a piece of wood hanging on a metal chain and an identical material dispenser as used in rearing pens (see description below). All pens were continuously numbered throughout all trials with unique PenIDs. Pigs had *ad libitum* access to water from four nipple drinkers per pen and to pellet concentrate by one single feeder (IVOG-feeding station, Hokofarm Group, Netherlands) per pen. The feed composition changed after the pigs reached an average weight of 80 kg (after approximately six weeks of fattening; see S2 Table). At approximately eleven weeks of age (i.e., after rearing), pigs were equipped with a LF-RFID ear tag (one per pig) for individual detection at the feeding station during fattening. All fattening pigs were treated identically among all trials except for the contents of the material dispensers (see Table 1). The total number of weeks of data collection during fattening varied depending on the underlying study but was always available from the beginning of fattening and for a period of at least nine weeks thereafter, which is why this period of data collection was uniformly used for the analysis of exploration and feeding data (i.e., UHF and LF-RFID data).

### Material dispenser and enrichment materials

During all trials from the four considered studies [11, 12, 29, 33], pigs had access to various plant-based enrichment materials via a material dispenser (Table 1; Fig 3). Therefore, all rearing and fattening pens were equipped with a material dispenser that consisted of a 100 cm high storage tube with a diameter of 25 cm. To create a rooting area, a 10 cm high cement ring with an inner diameter of 63 cm was installed around the storage tube. Underneath the storage tube and the cement ring, a mat was installed to prevent the enrichment material from falling through the slatted floor. A stock of enrichment material was filled into the storage tube, and the animals were given *ad libitum* access to the material through a variable adjustable gap between the storage tube and the mat on the floor by moving a 25 cm long, 5 cm wide and 0.8 cm thick plastic bolt with balls (diameter 5.5 cm) at both ends. By moving the plastic bolt, the pigs were able to move the enrichment material stored in the storage tube and obtain access to it (see S1 Video). The offered enrichment material and the intervals of changing the material differed between the studies (see Table 1).

### Data analysis for the retrospective evaluation

#### Exploration of the material dispenser

Each material dispenser was equipped with an UHF-RFID antenna (Kathrein, MIRA ETSI, Kathrein Solutions GmbH, Ismaning, Germany) to record the exact start and end times each individual pig stayed at the material dispenser [11]. This recorded time is referred to below as the exploration duration. The exploration durations for which each pig stayed at the material dispenser were automatically calculated (end time minus start time of stays) and stored in a database via a software application (Phenobyte GmbH & Co. KG, Ludwigsburg, Germany) that allowed us to assign each pig (AnimalID) to the corresponding pen (PenID). The material dispenser was validated in Kauselmann et al. [11].

#### Feeding data

Each single feeder (0.9 m × 0.6 m) of the fattening pens only recorded the exact duration (i.e., start and end time) of stays of each pig at the feeder (referred to below as feed consumption). For this purpose, the integrated LF-RFID system recorded the time when the pigs’ head moved into the feeding station and thus the LF-RFID tag in the pigs’ ear was detected. The difference in the weight of the feed in the trough at the start of the detected feed consumption event minus the weight of the feed in the trough at the end of the detected feed consumption event was recorded as the amount of feed consumed by a respective pig. The data were stored via an included software application. By adjusting the width of the feeder as the pigs grew, it was ensured that only one animal at a time had access to the trough of the feeder.

#### Tail biting outbreaks and tail scoring

All animals were visually inspected during daily routines. In case of tail biting, additional enrichment material was offered (see below), and observations of the pigs were intensified. The identified tail biters and severely bitten pigs were immediately removed from the group and kept in separate pens. Bitten pigs were closely observed and received individual medication depending on the severity of the tail injuries.

A tail biting outbreak in a pen was defined as the day when actions against tail biting had to be applied (for an overview, see S3 Table). This was the case when pigs were observed directly biting the tails of their pen mates or when bleeding tails were observed during the daily controls. In cases where tail biters could be identified, they were removed from the pen, and additional enrichment, i.e., paper bags, mineral feed, rolled oats and/or zeolite, was offered. Tail biting outbreaks (i.e., removed biters, days of tail biting outbreak, additional offered enrichment) were documented, and the days prior to the day of each tail biting outbreak within a pen were continuously numbered backwards and assigned to the respective pen (e.g., day 1 = one day prior to the tail biting outbreak, day 2 = two days prior to the tail biting outbreak, etc.).

At the end of rearing, each pig was scored according to the “Deutscher Schweine-Boniturschlüssel” [35] to record tail length losses in five categories: (0) full/natural length; (1) length loss up to one-third, i.e., we also included individuals with only minimal losses at the tassel; (2) reduction in tail length from at least one third to two-thirds; (3) length loss more than two-thirds; and (4) total loss with a maximum tail length of 1 cm remaining. However, a score of 4 was never recorded for any of the pigs during rearing or fattening.

### Statistical analysis

Since different subsets of the animals were considered for analyses in rearing and fattening (see Fig 1), all the data were analysed separately for rearing and fattening. All the statistical analyses were performed with the software R version 3.3.1 [36] and the package “nlme” [37]. The alpha level was set at 0.05 for all the statistical analyses. Given that this retrospective analysis was of an exploratory nature, we omitted p-value corrections for multiple testing. Each pig included in the analysis was assigned to one of three categories (in the following labelled as “CatPig”), i.e., tail biter, victim or neutral pig. Depending on the tail length score at the end of the respective production period (i.e., separately for rearing or fattening), pigs were assigned as victims, i.e., pigs with a tail length loss (score > 0), or neutral pigs, i.e., pigs without a tail length loss (score = 0). When a pig was observed while biting the tail of another pig, it was classified as a tail biter (there was no further classification of pigs classified as tail biters into victim or neutral pig–even if they had tail lesions). This categorization resulted in unbalanced sample sizes in the three categories, as only a smaller number of tail biters could have been individually identified. Days of tail biting outbreaks, when individual identified biters were removed from the groups, were not considered for statistical analyses. Furthermore, missing data due to technical issues were not considered for the statistical analyses.

We summed the recorded exploration duration at the material dispenser for each pig and day and used a linear mixed effect (LME) model for statistical analyses. Therefore, exploration data were log-transformed (log(x+1)) to achieve a normal distribution of the residuals, which was visually inspected and confirmed by q-q plots. The LMEs included the exploratory variables (i) CatPig (3-level factor: tail biter, victim or neutral pig), (ii) sex (2-level factor: female or castrated male), (iii) day prior to tail biting outbreak and (iv) two-way interactions between all the exploratory variables. Given that pigs within the same pen were not independent of each other, AnimalID and PenID were considered as nested random factors.

Furthermore, feed consumption, feeding duration and number of visits at the single feeder of the fattening pigs were calculated for each fattening pig and day. When incorrect weighing for feed consumption occurred, i.e., negative values of consumed feed occurred, these data were not considered for the statistical analyses (2.2% of the data points were affected). In the case of negative amounts of feed consumption (mostly very small values of -0.01 kg), the associated data points (i.e., frequency and duration of feed consumption) were also removed from the dataset. The negative values could not be attributed to technical defects or other plausible explanations (saliva from the pigs that possibly got into the trough, and the animals leaving the feeder without consuming feed, causing the final weight in the trough to be greater than the initial weight, resulting in negative values). The data of the amount and frequency of feed consumption were sqrt-transformed (sqrt(x+1)) to achieve a normal distribution of the residuals, which was visually inspected and confirmed by q-q plots. The LMEs used to analyse the effects on a) the amount of feed consumption, b) feeding duration and c) the number of visits at the feeder separately included the following exploratory variables: (i) CatPig (3-level factor: tail biter, victim or neutral pig), (ii) sex (2-level factor: female or castrated male), (iii) day prior to the tail biting outbreak and (iv) two-way interactions between all the exploratory variables. AnimalID and PenID were considered as nested random factors.

For pigs with birth weights available (i.e., 120 pigs in rearing and 58 pigs in fattening), daily weight gains during suckling were calculated as [(weaning weight-birth weight)/number of suckling days]. We used separate LMEs for rearing and fattening to analyse a) weight at birth, b) weaning weight and c) daily weight gain during suckling. Each model contained the exploratory variables (i) Cat-Pig (3-level factor: tail biter, victim or neutral pig), (ii) sex (two-level factor: female and castrated male) and (iii) two-way interactions between both explanatory variables. Data on daily weight gains during suckling and weaning weights were log-transformed (log(x)) to reach a normal distribution of the residuals, which was visually inspected and confirmed by q-q plots. The model used to analyse daily weight gain during suckling additionally included birth weight as an explanatory factor to control for variation at the beginning of the considered growth period.

For fattening, in addition, d) weight at the end of rearing, i.e., the weight with which they started the fattening period, and e) daily weight gain during rearing [(weight at the end of rearing-weaning weight)/days of rearing] were analysed with the same exploratory variables in the models (CatPig, sex and two-way interactions), and for weight gain during rearing, additionally weaning weight was considered as explanatory factor. AnimalID and PenID were considered as nested random factors for all analyses in rearing and fattening. In the case of significant differences, post hoc pairwise comparisons were performed via pairwise t-tests.

All the models used for the statistical analyses are summarized in the electronic supplement (see S1 File). Furthermore, all the data used for these analyses are provided at https://doi.org/10.6084/m9.figshare.26028748 (rearing) and https://doi.org/10.6084/m9.figshare.26028742 (fattening).

## Results

### Exploration duration during rearing

**Fig 4:**
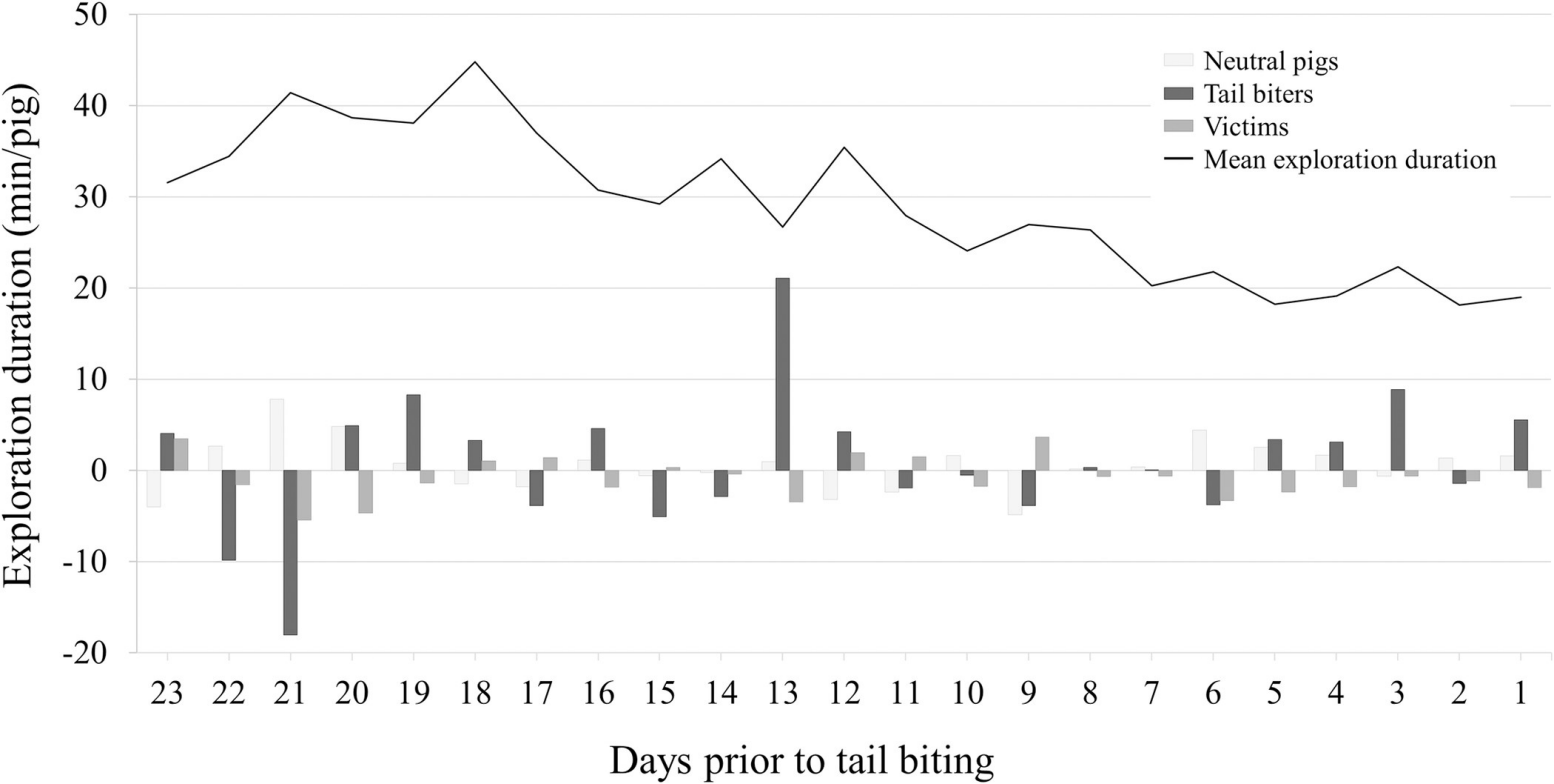
Mean duration of exploration per pig and day at the material dispenser on the days prior to tail biting during rearing (line); for visual representation, the deviation of the mean duration of exploration from the mean duration of neutral pigs (light grey bars), tail biters (black bars) and victims (dark grey bars) is shown.

Exploration duration at the material dispensers during rearing tended to be affected by CatPig, i.e., tail biters, victims and neutral pigs (LME, factor CatPig, F_2, 179_ = 2.61, P = 0.076). Victims spent less time at the material dispenser (22.0 min/pig and day ± 29.6 min/pig and day SD) compared to tail biters (29.4 min/pig and day ± 33.6 min/pig and day SD) and neutral pigs (26.3 min/pig and day ± 32.4 min/pig and day SD) (pairwise t-test: P < 0.001 for both comparisons). No significant difference was found between tail biters and neutral pigs (pairwise t-test: P = 0.14). No effect on exploration duration was found regarding the sex of the pigs (LME, factor sex, F_1, 179_ = 2.18, P = 0.14). The day prior to the tail biting outbreak had an effect on the exploration duration of the piglets (LME, factor day prior to tail biting, F_22, 3768_ = 8.99, P < 0.0001; Fig 4 and S4 Table). Highest exploration durations were observed on days 21 to 17 prior to an observed tail biting event, and lowest exploration durations were observed on days 7 to 1 prior to tail biting (S4 Table). There were also significant interactions between the day prior to tail biting and CatPig (LME, day prior to tail biting*CatPig, F_44, 3768_ = 1.78, P = 0.001). The exploration duration of the tail biters temporarily increased on day 13 (55.75 min ± 69.21 min SD) and on day 3 (35.12 min ± 55.27 min SD) before a tail biting outbreak was observed, whereas the exploration duration of the victims and neutral pigs remained at a lower level (day 13: victims: 23.21 min ± 26.39 min SD; neutral pigs: 27.99 min ± 31.48 min SD; day 3: victims: 21.73 min ± 20.35 min SD; neutral pigs: 21.98 min ± 21.70 min SD). The interactions between CatPig and the sex of the pigs (LME, CatPig*sex, F_2, 179_ = 0.26, P = 0.77) and the day prior to tail biting and the sex of the pigs (LME, day prior to tail biting*sex, F_22, 3768_ = 0.74, P = 0.80) were not significant.

### Effect of weight during rearing

**Fig 5:**
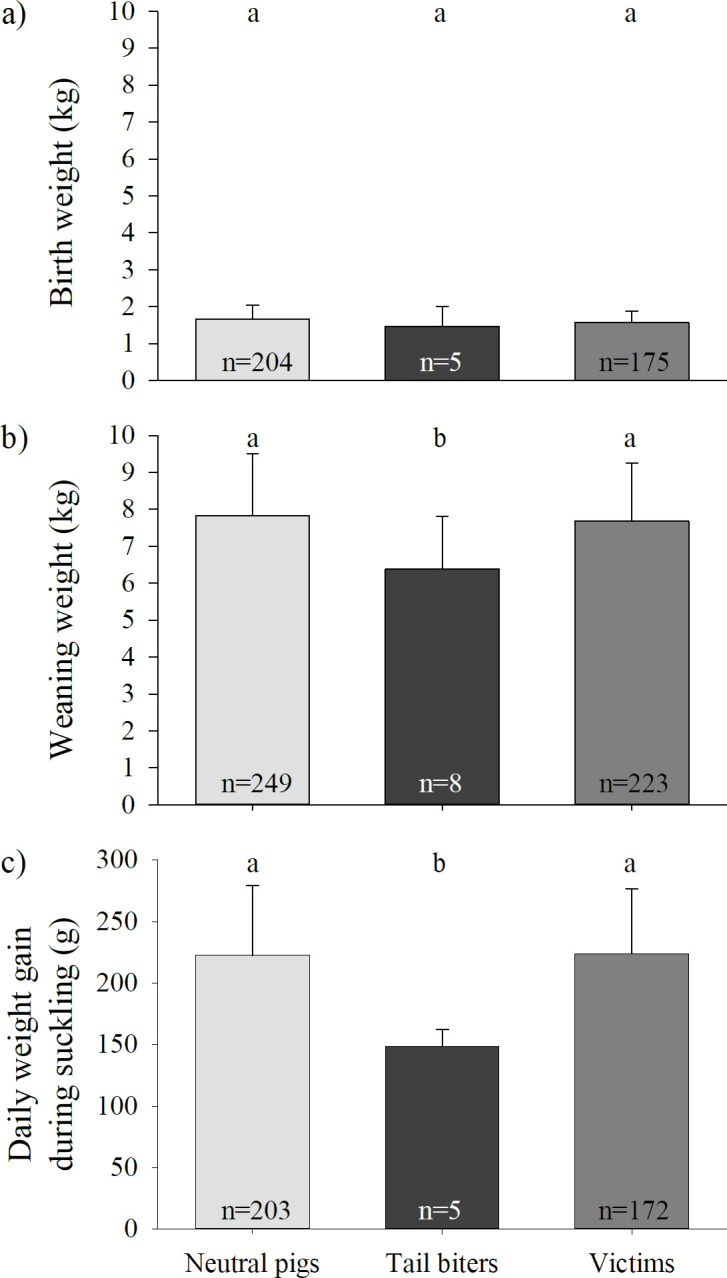
a) Mean birth weight, b) mean weaning weight and c) mean daily weight gain during suckling (±SE) of pigs classified as neutral pigs (light grey), tail biters (black) and victims (dark grey) during rearing. Different letters indicate significant differences among the CatPig classifications (neutral pig, tail biter or victim).

There was neither a difference in birth weight between tail biters, victims and neutral pigs (LME, factor CatPig, F_2, 110_ = 1.11; P = 0.33; Fig 5A; S1a Table) nor in sex (LME, factor sex, F_1, 110_ = 0.10; P = 0.75), and the interaction between CatPig and sex was not significant (LME, CatPig*sex, F_2, 110_ = 1.71; P = 0.19).

However, there was a significant difference in the daily weight gain during suckling between tail biters, victims and neutral pigs (LME; factor CatPig, F_2, 109_ = 6.70; P = 0.0018; Fig 5C; S1a Table). Tail biters had lower daily weight gains during suckling (148.36 g/d ± 13.82 g/d SD) compared to victims (223.82 g/d ± 52.72 g/d SD) and neutral pigs (222.61 g/d ± 56.45 g/d SD) (pairwise t-tests: P < 0.01 for both comparisons). No significant difference was found between victims and neutral pigs (pairwise t-test: P = 0.91). Daily weight gains during suckling were not affected by sex (LME, factor sex, F_1, 109_ = 0.64; P = 0.43), but with increasing birth weight, the daily weight gains were higher during suckling (LME, factor birth weight, F_1, 109_ = 10.69; P = 0.0014). With respect to weight gain during suckling, there was no significant interaction effect between CatPig and sex (LME, CatPig*sex, F_2, 109_ = 0.27, P = 0.76).

There were differences in weaning weight between tail biters, victims and neutral pigs (LME, factor CatPig, F_2, 179_ = 4.07, P = 0.019; Fig 5B; S1a Table). Weaning weight of tail biters (6.38 kg ± 1.44 kg SD) was significantly lower compared to victims (7.68 kg ± 1.58 kg SD; pairwise t-test: P = 0.03) and neutral pigs (7.82 kg ±1.68 kg SD; pairwise t-test: P = 0.02). The weaning weights of the victims and neutral pigs did not differ from each other (pairwise t-test: P = 0.56). Weaning weights neither were affected by sex (LME, factor sex, F_1, 179_ = 0.86; P = 0.36) nor by the interaction between sex and CatPig (LME, CatPig*sex, F_2, 179_ = 1.67, P = 0.19).

### Exploration duration during fattening

Exploration duration at the material dispenser was neither affected by CatPig (LME, factor CatPig, F_2, 48_ = 0.12, P = 0.89) nor by sex (LME, factor sex, F_1, 48_ = 1.84, P = 0.18). The day prior to a tail biting outbreak had an effect on the exploration duration of pigs (LME, factor day prior to tail biting, F_22, 635_ = 6.04, P < 0.0001). Independent of CatPig highest exploration durations were recorded on day 19 (42.77 min ± 19.40 min SD; S4 Table) and day 18 (42.44 min ± 18.12 min SD) prior to a tail biting outbreak, followed by a decrease in exploration duration on day 17 (24.27 min ± 12.06 min SD). The exploration duration subsequently showed an inconsistent course. None of the interactions analysed were significant (LME, CatPig*sex: F_2, 48_ = 0.52, P = 0.60; CatPig*day prior to tail biting: F_44, 635_ = 0.72, P = 0.91; sex*day prior to tail biting: F_22, 635_ = 0.44, P = 0.99).

### Effect of weight during suckling and rearing on tail biting during fattening

**Fig 6:**
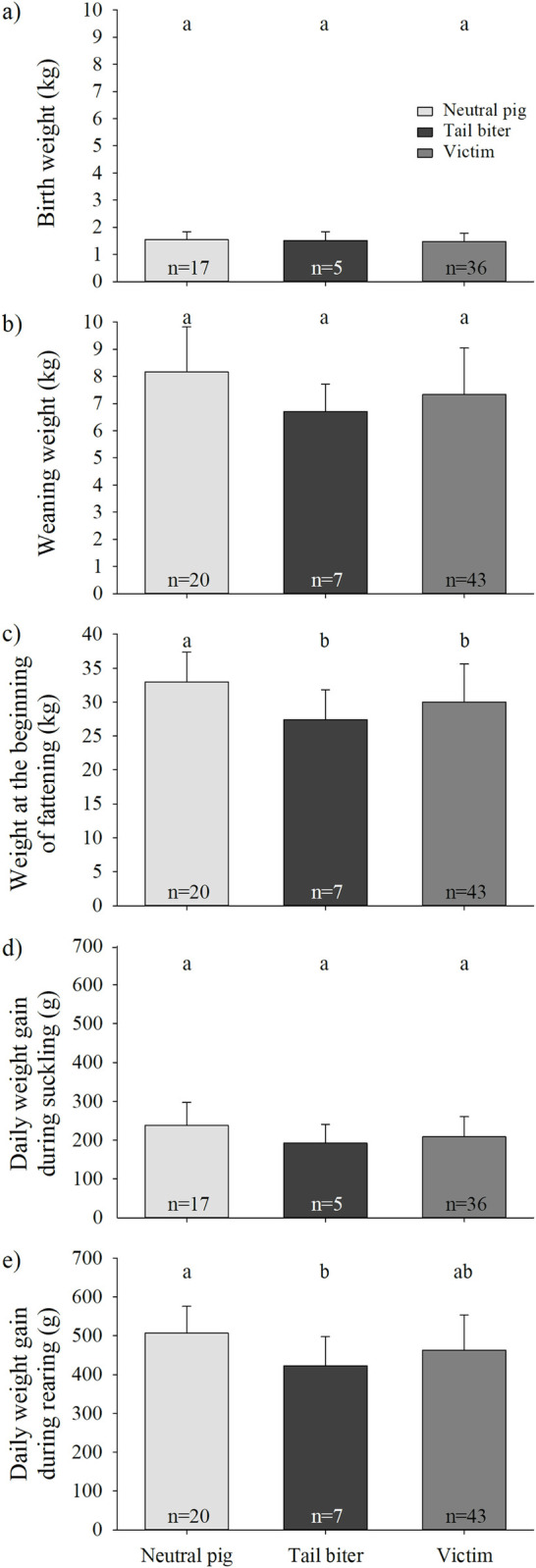
a) Mean birth weight, b) mean weaning weight, c) mean weight at the beginning of fattening, d) mean daily weight gain during suckling and e) daily weight gain during rearing (±SE) of pigs classified as neutral pigs (light grey), tail biters (black) and victims (dark grey) during rearing. Different letters indicate significant differences among the CatPig classifications (neutral pig, tail biter or victim).

For fattening pigs, there was neither a difference in birth weight between tail biters, victims and neutral pigs (LME, factor CatPig, F_2, 48_ = 0.21; P = 0.81; Fig 6A, S1b Table) nor in sex (LME, factor sex, F_1, 48_ = 0.02; P = 0.90). The weaning weight tended to affect CatPig (becoming a tail biter, victim or neutral pig) in fattening (LME, factor CatPig, F_2, 59_ = 2.72; P = 0.07; Fig 6B, S1b Table). Neutral pigs seemed to have higher weaning weights than tail biters and tended to have higher weaning weights compared to victims, but no obvious differences were apparent between tail biters and victims. There was no difference in weaning weight between sexes (LME, factor sex, F_1, 59_ = 0.51; P = 0.48). There was a difference in weight at the beginning of fattening between tail biters, victims and neutral pigs (LME, factor CatPig, F_2, 59_ = 3.60; P = 0.03; Fig 6C, S1b Table). Neutral pigs had higher weights at the beginning of fattening compared to tail biters (pairwise t-test: P = 0.02) and victims (pairwise t-test: P = 0.04), but no differences were found between tail biters and victims (pairwise t-test: P = 0.22). There was no difference in weight at the beginning of fattening between sexes (LME, factor sex, F_1, 59_ = 0.93; P = 0.34). Weight gain during suckling had no effect on becoming a tail biter, victim or neutral pig in fattening (LME, factor CatPig, F_2, 47_ = 2.43; P = 0.10; Fig 6D, S1b Table), and there were no differences between the sexes (LME, factor sex, F_1, 47_ = 0.94; P = 0.34). There was an effect of weight gain during rearing on becoming a biter, victim or neutral pig in fattening (LME, factor CatPig, F_2, 58_ = 4.19; P = 0.02; Fig 6E, S1b Table). Neutral pigs had higher weight gains during rearing compared to tail biters (pairwise t-test: P = 0.03) and tended to have higher weight gains during rearing compared to victims (pairwise t-test: P = 0.06), but no differences were found between tail biters and victims (pairwise t-test: P = 0.24). There were no differences in weight gain during rearing between the sexes (LME, factor sex, F_1, 58_ = 1.45; P = 0.23).

### Amount, duration and frequency of feed consumption during fattening

**Fig 7:**
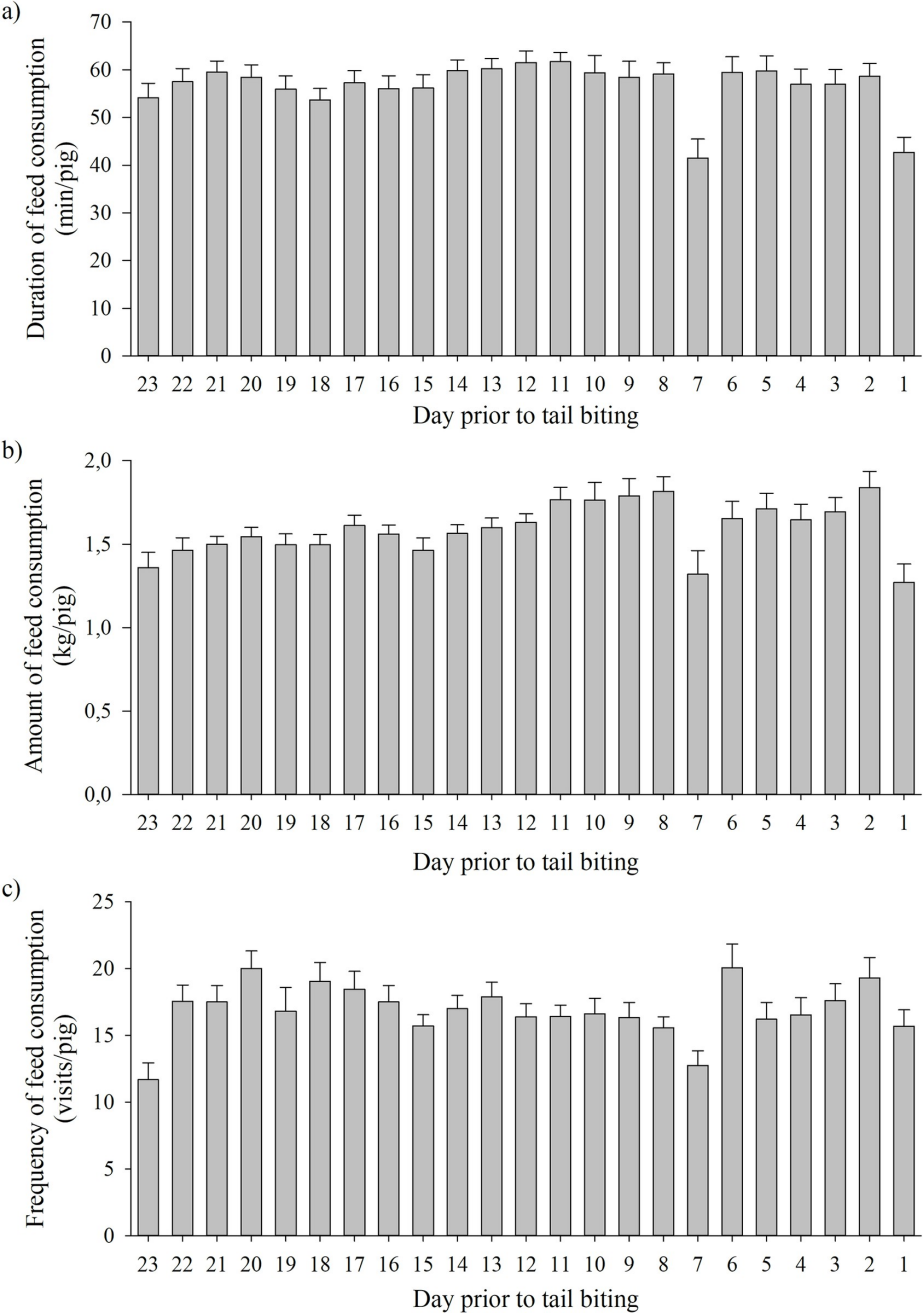
Duration (min/pig ± SD), amount (kg/pig ± SD) and frequency (visits/pig ± SD) of feed consumption on the days prior to tail biting during fattening.

The amount, duration and frequency of feed consumption was neither affected by CatPig (LME, factor CatPig_amount_: F_2, 59_ = 1.08, P = 0.35; factor CatPig_duration_: F_2, 59_ = 0.39, P = 0.68; factor CatPig_frequency_: F_2, 59_ = 0.21, P = 0.81) nor by sex (LME, factor sex_amount_: F_1, 59_ = 0.52, P = 0.47; factor sex_duration_: F_1, 59_ = 0.27, P = 0.61; factor sex_frequency_: F_1, 59_ = 0.03, P = 0.86). However, the days prior to a tail biting outbreak had an effect on feed consumption (LME, factor day prior to tail biting outbreak_amount_: F_22, 636_ = 13.25, P < 0.0001; factor day prior to tail biting outbreak_duration_: F_22, 636_ = 4.00, P < 0.0001; factor day prior to tail biting outbreak_frequency_: F_22, 636_ = 4.64, P < 0.0001; Fig 7). Amount and duration of feed consumption decreased on day 7 prior to tail biting, increased again (compared to day 7), and showed another decrease on day 1 prior to tail biting. Frequency of feed consumption showed an inconsistent course, also decreased on day 7 prior to tail biting and increased on day 6 prior to tail biting to a higher level than before. With respect to the duration of feed consumption, there was a significant interaction effect between sex and day prior to the tail biting outbreak (LME, factor sex*day prior to the tail biting outbreak, F_22, 636_ = 1.87, P < 0.01). Immediately before tail biting was observed (days 2 to 5 prior to tail biting), the castrated male pigs spent more time at the feeder, whereas three weeks prior to tail biting and on days 9 and 10 prior to tail biting, the duration of feed consumption of the female pigs was higher. All further two-way interactions between CatPig, sex and day prior to tail biting were not significant (data given in the electronic supplement; S5 Table).

## Discussion

### Exploration

In relation to tail biting outbreaks, we found certain changes in the temporal course of exploration duration at the material dispensers. In rearing pigs, these changes differed between tail biters, victims and neutral pigs (CatPig). However, no differences in exploration duration between CatPig were found in fattening pigs.

In rearing, there was a decrease in exploration duration at the material dispenser prior to tail biting. This finding seems to be in contrast to previous studies in which the activity of pigs increased prior to tail biting [20–23]. However, an overall increase in activity could indicate that pigs spend less of their time exploring, and both behavioural changes (increase in activity and decrease in exploration) may indicate an upcoming tail biting event. Decreased exploration durations over time can also be affected by the habituation of pigs to the offered enrichment materials [38, 39] or because exploration was reoriented from the material dispensers to the conspecifics (tails). However, it can be assumed that an upcoming tail biting event rather than habituation affected exploration durations, since days prior to tail biting outbreaks were numbered backwards for the analyses and the exploration duration was maintained or even increased over the entire period of the original studies considered here [11, 12, 29, 33].

Exploration duration was not associated with CatPig in fattening, whereas in rearing, CatPig tended to be linked to exploration duration, with victims showing lower exploration durations than tail biters and neutral pigs did. For this retrospective explorative analysis, no systematic behavioural observations with respect to tail biting were available, as it was not part of the original data collection in the studies [11, 12, 29, 33]. Thus, on the one hand, we likely underestimated the number of biters. On the other hand, we presumably falsely categorized some nonidentified biters as victims or neutral pigs, which might have affected the present results. Due to the overall small number of individual biters (i.e., 15, eight biters in rearing, seven biters in fattening), further experimental investigations with a focus on biters, apart from the present retrospective explorative analyses, are needed to support our results. Nevertheless, depending on the days prior to tail biting, differences between the pigs within the three CatPig categories were found. In rearing, biters showed short-term increases in exploration duration prior to tail biting, whereas victims and neutral pigs showed a more uniform course. Previous studies found that pigs show individual behavioural differences [40, 41] and differences in their reactions to stress in the “back test” [42, 43]. This finding might indicate different strategies by which pigs cope with stress [44]. However, there seem to be no consistent results suggesting a clear link between the coping type of pigs and their adaptation to stress [43, 45–47]. It is assumed that abnormal behaviours, i.e., tail biting, occur when pigs fail to cope with their housing environment [3, 48]. This may also have been the case in the current study and may have led to the increase in exploration duration of pigs becoming tail biters prior to a tail biting outbreak in rearing. Thus, detecting individual pigs that deviate from exploration behaviour towards rootable plant-based enrichment material within a group of pigs could be a promising measure for an early detection of tail biting during rearing in the future.

However, in this retrospective explorative analysis, different studies in which different enrichment materials were offered to the pigs through an identical material dispenser were considered. Furthermore, additional enrichment materials (i.e., ropes and wood) were offered whose use by the pigs was not recorded. Both could have interfered with the effect on the recorded exploration durations over time (i.e., prior to a tail biting outbreak). However, with respect to the additionally offered but not studied enrichment materials, the interference should be limited, as these materials were offered identically within all pens, both in all studies and within rearing and fattening. This could also explain why the data collected at the material dispenser were valuable for predicting tail biting outbreaks.

### Weight parameters

Some weight parameters differed in terms of whether pigs were categorized as tail biters, victims or neutral (CatPig) in rearing (i.e., weight gain during suckling and weaning weight) and fattening (i.e., weight at the beginning of fattening, weight gain during rearing).

In rearing, there was no covariation between birth weight and CatPig, but weight gain during suckling and weaning weight (weight at the age of four weeks) were significantly related to CatPig in rearing. Tail biters identified in rearing turned out to have lower weight gains during suckling and lower weaning weights compared to neutral pigs and victims in rearing. Therefore, the likelihood of estimating whether an individual pig will become a tail biter by using their weight seems to be possible at the earliest during or after the suckling period. Accordingly, Zonderland et al. [23] also found that biters had lower start weights (weight at the age of four weeks) compared to tail biting victims. Beattie et al. [27] differed between pigs that showed more or less than 1.5% tail biting of the observed time and found that pigs performing >1.5% tail biting had lower daily weight gains during suckling, lower weights at weaning (with an age of 26 days) and lower weights seven days after weaning compared to pigs performing <1.5% tail biting. They suggested that nutritional deficiency during suckling may increase foraging behaviour (i.e., chewing ropes), which could result in tail biting that may persist even after weaning [27]. In contrast, Ursinus et al. [49] found that pigs that showed a high degree of tail biting at 6–8 weeks of age had higher weaning weights and higher average daily weight gains during suckling compared to pigs that do not show tail biting. However, these authors also suspected that tail biting could be related to the metabolic requirements of fast-growing pigs [49]. Very little is known about the effects during suckling on tail biting in pigs (i.e., birth order, litter size, suckling order/position at the teat, weaning weight and age at weaning). EFSA [50] reported that the stress of weaning decreases exponentially with increasing age of piglets (i.e., weaning age between 7 and 35 days). However, there are not enough data available to assess the effect of weaning age on pigs weaned at an age older than 28 days. Interestingly, Mason [51] showed that early weaning in mink (at seven weeks of age) resulted in increased tail biting compared with late weaning (at eleven weeks of age). Thus, weaning at an age older than four weeks could also have a reducing effect on tail biting in pigs by potentially allowing piglets to compensate for nutrient or social deficits over a longer period of time. This is an especially interesting explanation when considering that piglets in a seminatural environment are weaned approximately 60 to 100 days after birth [52]. However, further investigations in pigs are needed to specify the postnatal effects of birth, suckling and weaning age on tail biting and becoming a tail biter later in life.

In fattening, there was a covariation between CatPig and both weight at the beginning of fattening and daily weight gain during rearing. Neutral pigs had higher weights at the beginning of fattening compared to tail biters and victims, and daily weight gain during rearing was also higher in neutral pigs compared to both other CatPig (i.e., victims or tail biters). Thus, similar to the results for rearing pigs, it seems to be possible to predict the role of a pig (i.e., CatPig) during a tail biting event in fattening to a certain degree. However, the earliest this seems to be possible is during or after the rearing period. Furthermore, no differentiation between tail biters and victims seems to be possible, which could indicate that both CatPig have experienced stress (either prior to or due to tail biting). Tail biting can cause weight loss in pigs [53, 54] and mainly occurs for the first time during rearing [e.g. 55, 56]. Thus, weight loss due to tail biting in rearing could have also caused changes in weight, which could be a trigger for abnormal behavioural tendencies during fattening.

No long-term effects of birth weight and weight gain during suckling were found in fattening with respect to CatPig. However, there was a tendency in weaning weight and neutral pigs seem to have higher weaning weights compared to tail biters and victims. Thus, similar to the results for rearing pigs, weight parameters that date back several weeks no longer seem to have an effect on CatPig.

The results indicate that homogeneous and high weights and proper growth within litters seem to reduce the risk of tail biting later in life.

### Amount, duration and frequency of feed consumption

The amount, duration and frequency of feed consumption in fattening pigs were linked to the day prior to tail biting. However, there was no association from CatPig to measures of feed consumption during fattening. Feeding data (i.e., duration, amount and frequency of feed consumption) were only available for fattening and showed differences between the days prior to tail biting. Duration of feed consumption was constant on the days prior to tail biting, with the exception of day 7 and day 1 prior to tail biting, when durations of feed consumption decreased compared to the previous day. After day 7, the duration of feed consumption increased again on day 8. Similarly, the amount of feed consumption decreased on day 7 and day 1 prior to tail biting compared to the previous day and following day. The frequency of feed consumption showed a more inconsistent course compared to the duration and amount of feed consumption. However, the frequency of feed consumption also decreased on day 7 compared to day 9 prior to tail biting. As reduced health or welfare can induce a reduction in feed intake of pigs [57, 58], it can be assumed that pigs can also show a reduced feed intake due to a tail biting outbreak. The current study shows that a change in the feeding behaviour of pigs occurred both immediately before the outbreak of tail biting (i.e., one day prior to detection of tail biting) and a few days before the tail biting outbreak (i.e., seven days prior to tail biting).

In addition, during and even before tail biting becomes visible, victims (i.e., pigs with wounds or blood at the tail) show reduced feed intake compared with pigs without signs of biting at the tail [24]. Wallenbeck and Keeling [25] compared data of electronic feeders between victims (i.e., pigs that have to be treated or culled as a consequence of tail damage from tail biting), nonvictims (i.e., those not treated or culled because of tail biting) from the same pen and control pigs from pens without registered tail damage and found that five to two weeks before the first measures against tail biting were taken, victims showed a higher number of visits at the feeder compared to nonvictims and control pigs. Thereafter, approximately one week before measures against tail biting were taken, the number of visits at the feeder decreased in victims [25]. Thus, a decrease in feed intake (amount, duration or frequency) can indicate an upcoming tail biting event. In our study, no differences between biters, victims and neutral pigs (CatPig) were found, which was probably partly confounded by a certain number of biters that were not identified in the original studies (see Section 4.1).

In general, the amount of consumed feed during fattening slowly increased over time (days prior to tail biting), whereas the duration of feed consumption remained at an almost constant level (with the exception of days 7 and 1 prior to tail biting), and the frequency of feed consumption somewhat varied between the days prior to tail biting. The increasing amount of feed consumption was to be expected considering the increasing age of the fattening pigs [e.g. 59, 60]. However, in contrast to our results, Hoy et al. [60] found a decrease in the number of visits at the feeder from the beginning to the end of fattening, whereas the duration per stay at the feeder and the amount of feed consumption increased. In our study, only three weeks of fattening were considered at different time intervals for statistical analysis (counted backwards from the day of the tail biting outbreak), which probably obscured the temporal effects on the amount, duration and frequency of feed consumption. Furthermore, besides tail biting, other factors can influence the feed intake of pigs [61], e.g., the composition of the offered diet [e.g. 62]. In the present study, the feed composition changed when fattening pigs reached an average weight of approximately 80 kg (after approximately six weeks of fattening). However, in our study, all the biters were identified before the feed composition changed. Thus, by additionally considering the days prior to a tail biting outbreak for analyses, behavioural changes in feed consumption due to the upcoming tail biting outbreak rather than a change in feed composition affected the changes in feeding behaviour.

On the basis of our retrospective exploratory evaluation carried out here, the change in the feeding behaviour of fattening pigs on day 7 prior to tail biting cannot be clearly explained with a biologically relevant hypothesis. Thus, to discern whether this phenomenon is biologically significant or just noise in the data, further studies are needed.

### Overall discussion

Overall, the data on the exploration duration of pigs (in rearing and fattening) and feeding behaviour (in fattening) could be suitable indicators for developing early prediction measures of tail biting on pen level in future studies. Both methods would require the installation of material dispensers and/or feeding stations for individual animal identification (e.g., via RFID systems). On the other hand, the use of weight data, which are already routinely collected on some farms and would therefore result in lower financial and labour costs, also seems to be suitable for the early detection of individual pigs involved in tail biting. Thus, depending on the investigations (i.e., pen or individual level) and the financial/labour resources, it should be decided which method is the most suitable for the respective farm. Thus, our retrospective exploratory study could contribute to identifying suitable measures for the early detection of tail biting. Future studies shall ensure that all biters are systematically and individually identified. Once predictors are identified, they should also be validated in an experimental study in which predictors are tested to verify their validity.

## Conclusion

In this retrospective explorative analysis, automatically recorded exploration durations at a material dispenser and feed intake of rearing and fattening pigs, as well as weight parameters from birth to fattening, were considered to investigate whether these data are associated with tail biting and thus have the potential to be used for early detection of tail biting in rearing and fattening in future studies. In rearing, exploration duration could be a suitable indicator for the early detection of tail biting outbreaks within a pen and the identification of individual pigs that may become tail biters in an upcoming tail biting event. Furthermore, for early detection of tail biters during rearing, weight parameters during the suckling period (weight gain during suckling and weaning weight) seem to have great potential. Here, a certain potential for prevention may exist by ensuring proper and homogenous weight gains within litters. In fattening, exploration duration and feed intake (amount, duration and frequency of feed consumption) could be suitable indicators for the early detection of tail biting outbreaks within a pen but are less suitable for identifying tail biters prior to a tail biting outbreak. Furthermore, weight parameters in rearing (i.e., weight at the beginning of fattening and weight gain during rearing) seem to have great potential for the early detection of tail biting during fattening. However, weight parameters during the suckling period do not seem to be suitable for identifying individual pigs that may become tail biters in an upcoming tail biting event during fattening. However, owing to the limited number of individuals identified as biters in this retrospective analysis and the fact that data on feed consumption were collected only during fattening, further investigations are needed to confirm our findings. Nevertheless, the parameters evaluated in our retrospective analysis highlighted that exploration behaviour and weight might be candidate measures for future early prediction of tail biting during rearing.

## Supporting information

S1 Table Weight measures of pigs included in the analyses in a) rearing and b) fattening depending on the three categories of pigs (CatPig: 0 = neutral pigs; 1 = tail biters; 2 = victims). Birthweight, weaning weight and daily weight gain during suckling are shown for the three categories of pigs (CatPig) in rearing; birthweight, weaning weight, daily weight gain during suckling and daily weight gain during rearing are shown for the three categories of pigs (CatPig) in fattening.
(TIF)

S2 Table Composition of the two feed rations during rearing (change of feed after two 1 weeks of rearing) and fattening (change of feed at an average weight of 80 kg). (TIF)

S3 Table Overview of days of tail biting outbreaks within the rearing and fattening pens considered for the retrospective analyses. (TIF)

S4 Table Mean exploration duration in minutes (±SD) per pig and day prior to tail biting outbreak during rearing and fattening. (TIF)

S5 Table Statistical analyses of the effect of CatPig (neutral pig, tail biter or victim), day prior to tail biting and sex on the amount, duration and frequency of feed consumption in fattening pigs. (TIF)

S1 Video Rearing pigs move the plastic bolt with balls at the end to access the enrichment material stored in the material dispenser. (MP4)

S1 File Models used for statistical analyses separately for rearing and fattening. (TIF)
