# Supplementary figures and images for: Retrospective exploratory evaluation of individual pigs’ behaviour involved in tail biting during rearing and fattening
Source: PLoS One. 2025 Jan 13;20(1):e0316044. doi: 10.1371/journal.pone.0316044 (PMC11730382; doi:10.1371/journal.pone.0316044)

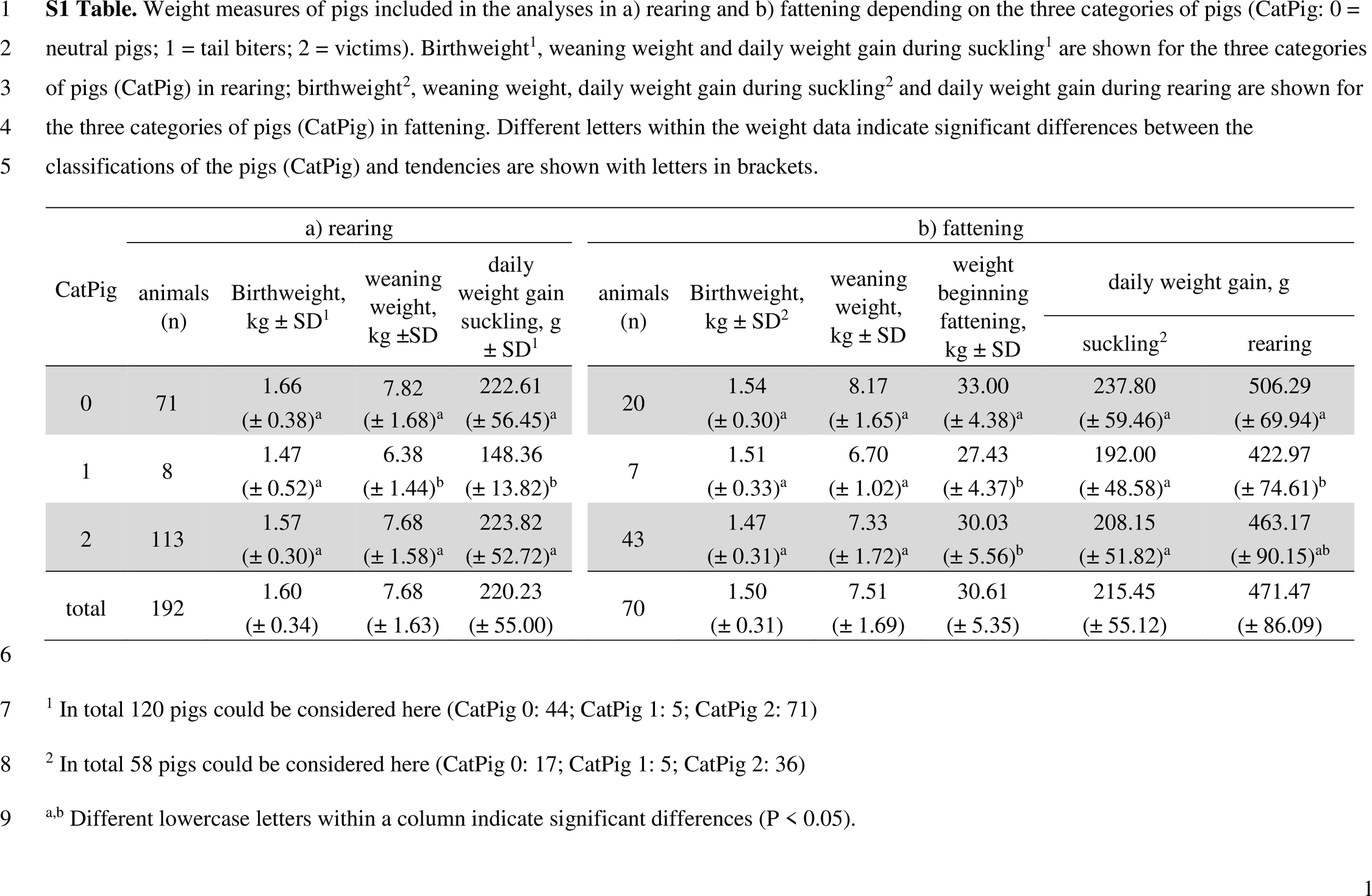

Supplement: S1 Table — Birthweight, weaning weight and daily weight gain during suckling are shown for the three categories of pigs (CatPig) in rearing; birthweight, weaning weight, daily weight gain during suckling and daily weight gain during rearing are shown for the three categories of pigs (CatPig) in fattening. (TIF) [file pone.0316044.s001.tif]

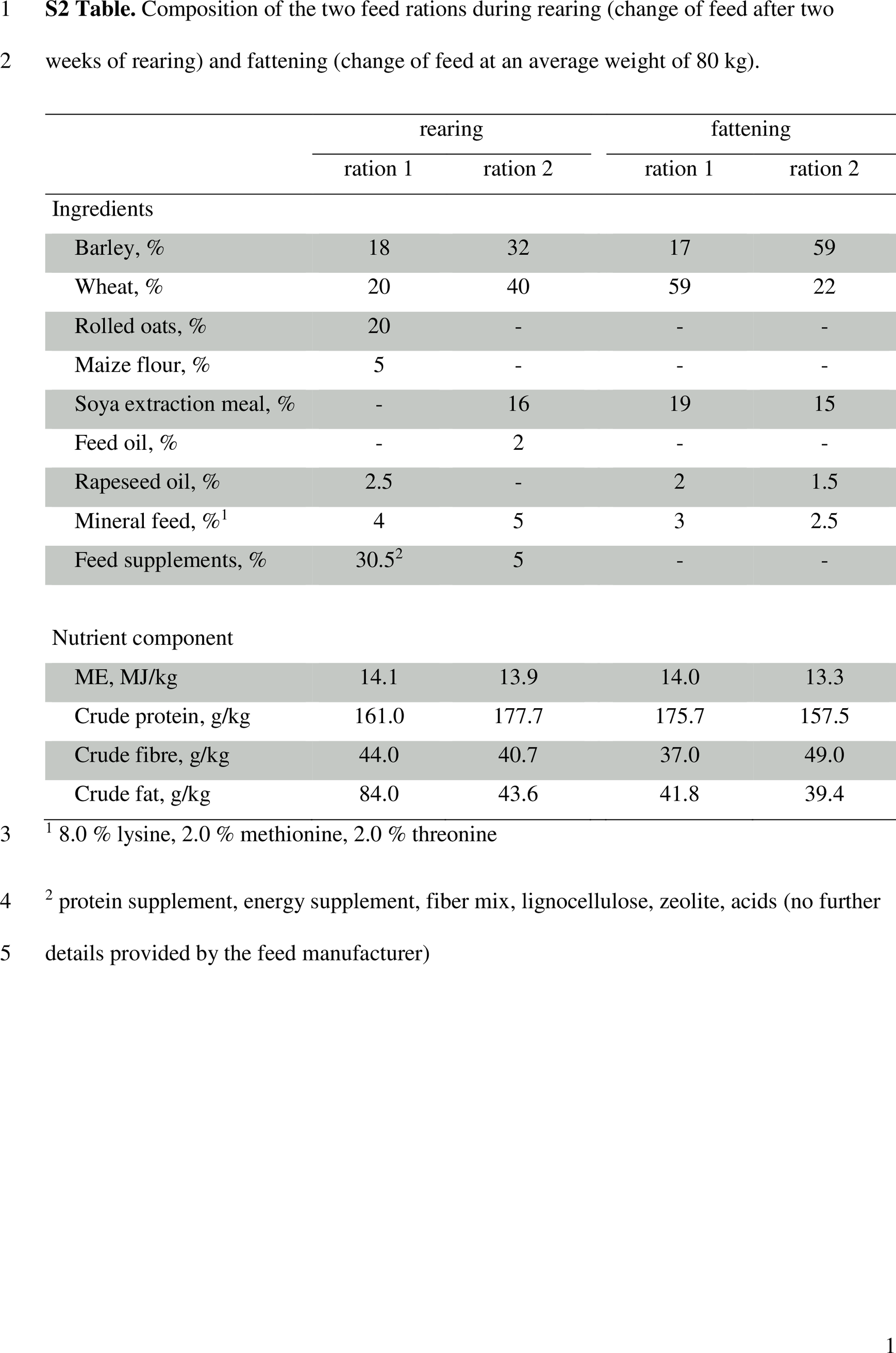

Supplement: S2 Table — (TIF) [file pone.0316044.s002.tif]

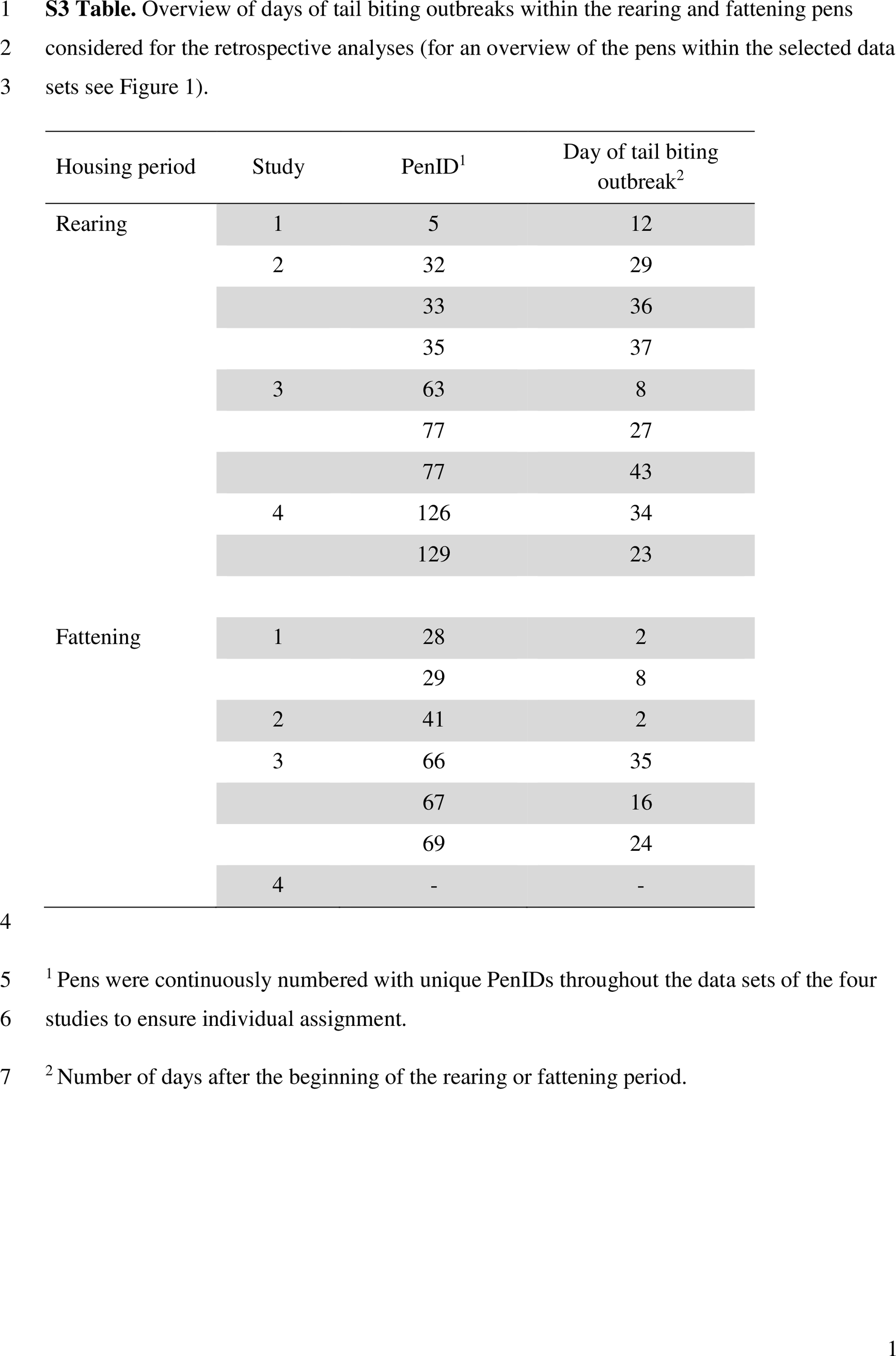

Supplement: S3 Table — (TIF) [file pone.0316044.s003.tif]

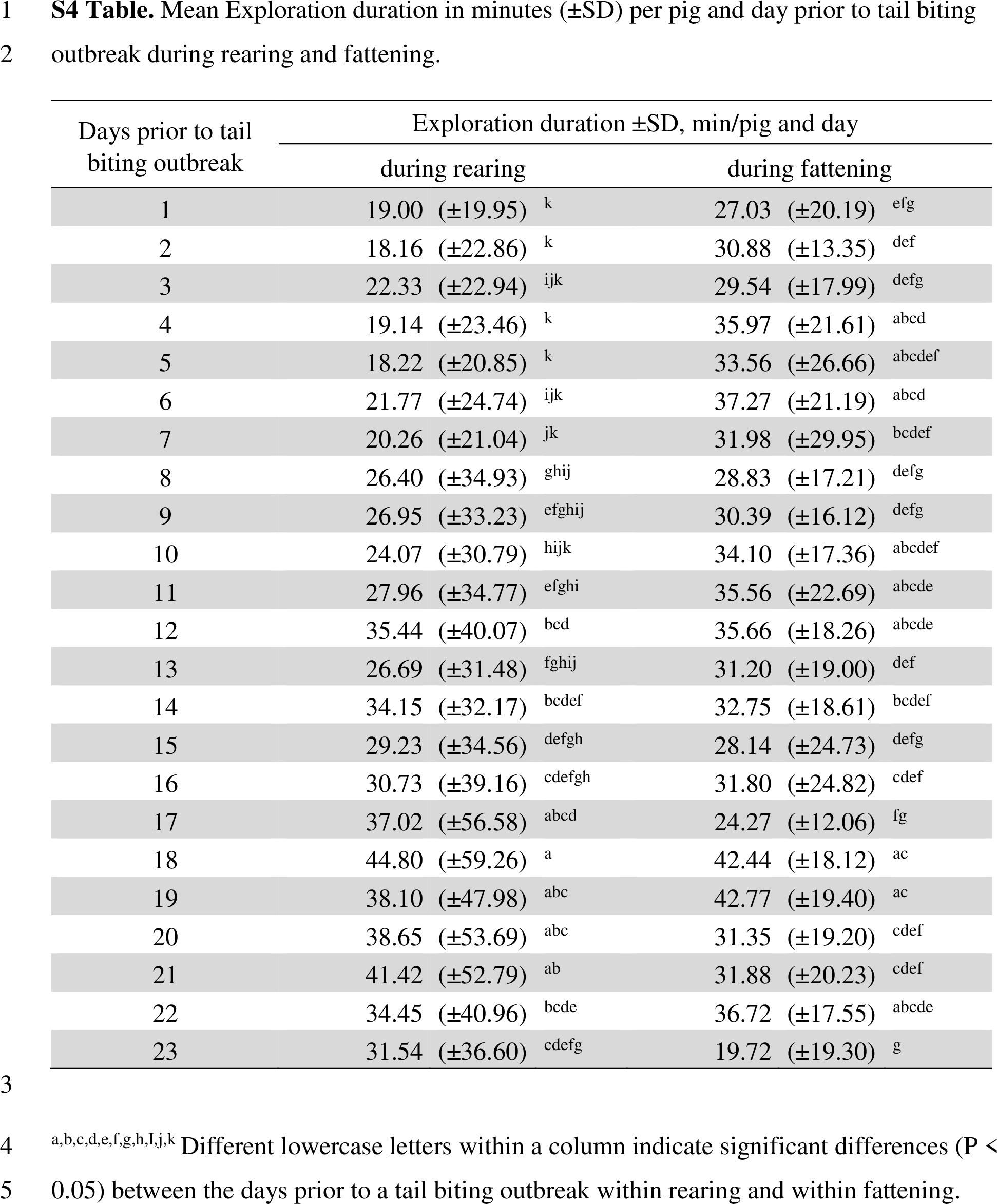

Supplement: S4 Table — (TIF) [file pone.0316044.s004.tif]

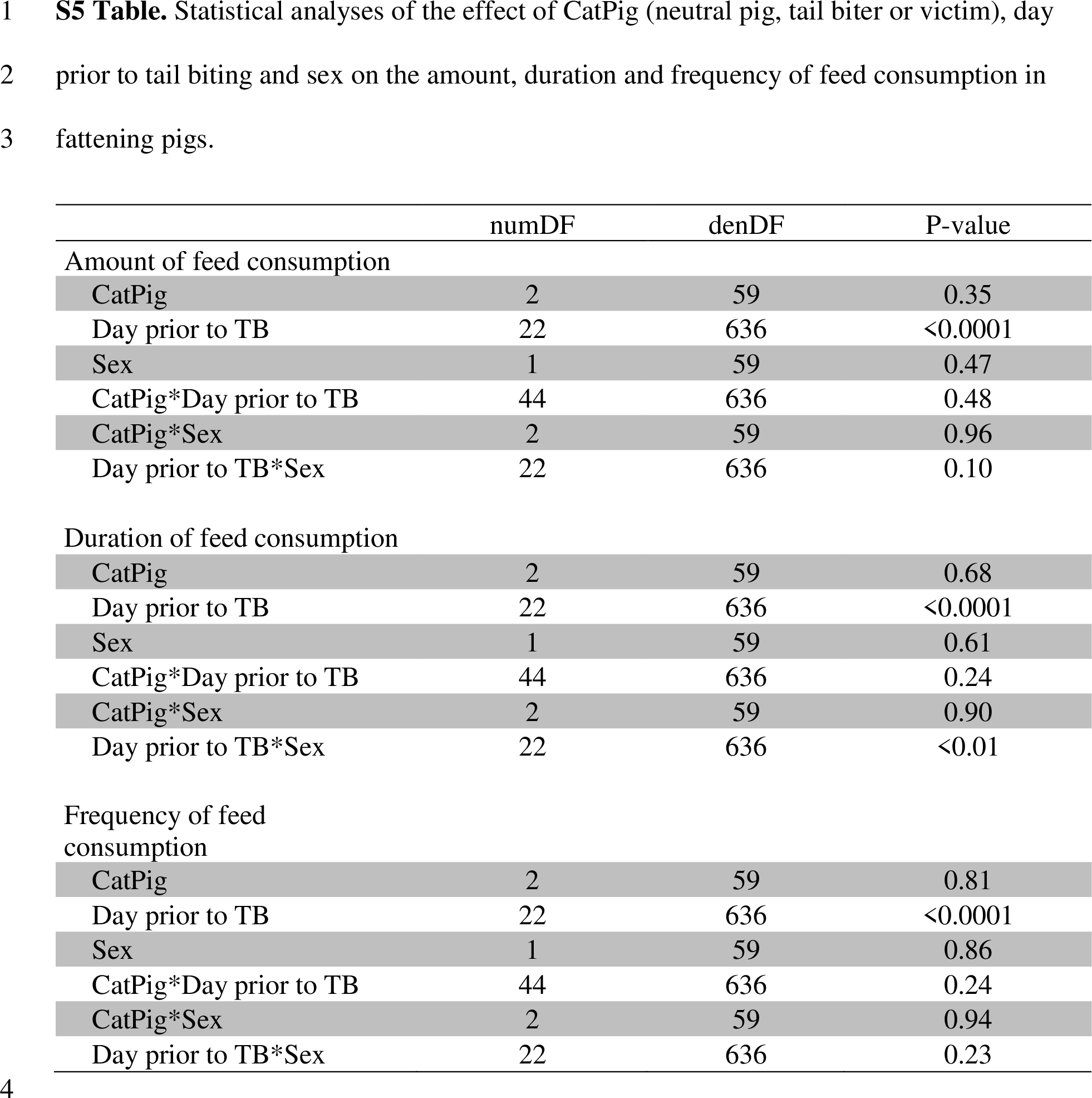

Supplement: S5 Table — (TIF) [file pone.0316044.s005.tif]

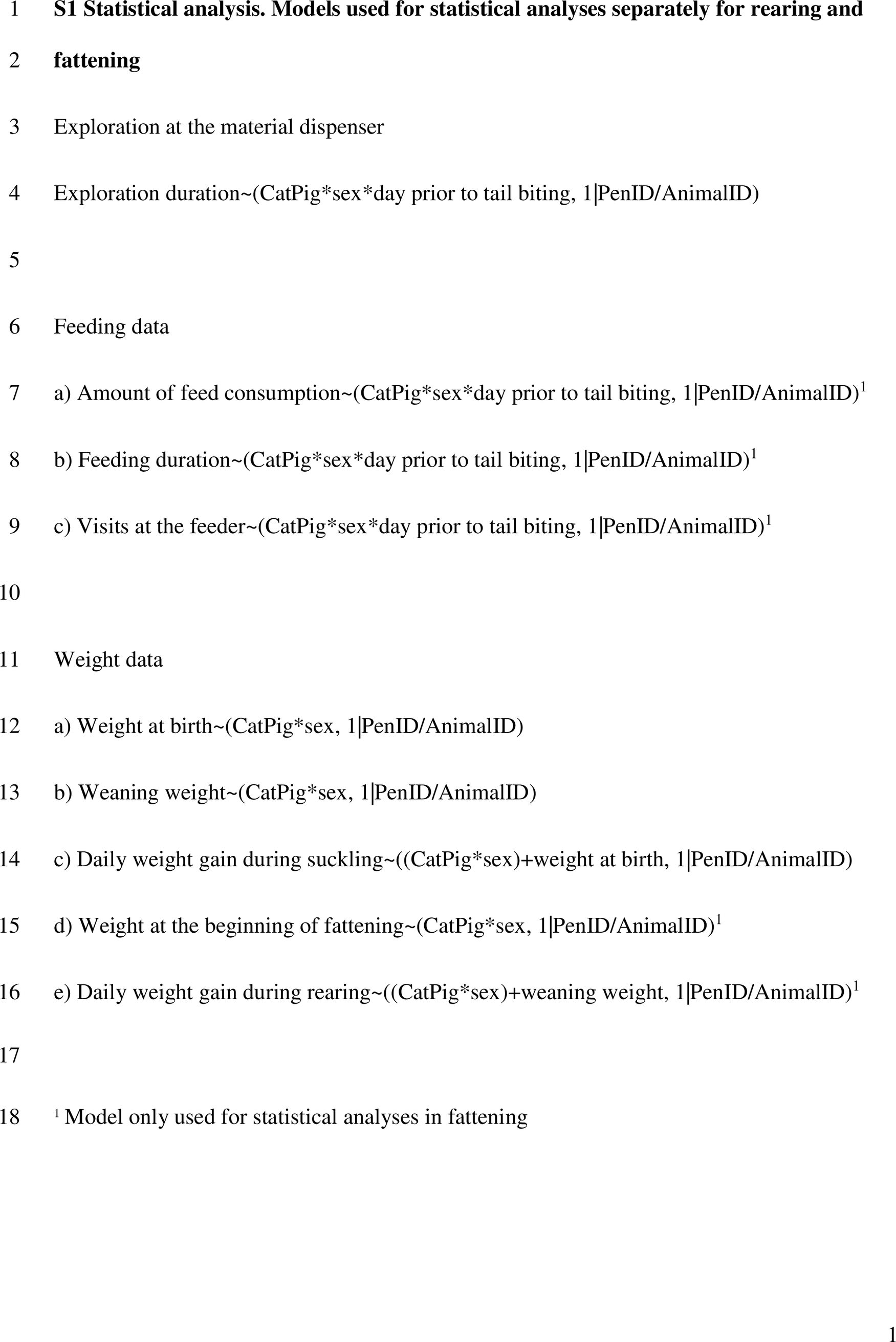

Supplement: S1 File — (TIF) [file pone.0316044.s007.tif]
